# Mechanistic Insights Into the Cardioprotective Effects of Modern Glucose-Lowering Drugs in Type 2 Diabetes: A Systematic Review

**DOI:** 10.7759/cureus.93284

**Published:** 2025-09-26

**Authors:** Mounica Ratnala, Loveleen K Johal, Zulaihat F Galadima, Fatima F Janjua, Katherine S Trejos Guzman, Kirshan Lal, Maryam Fatima, Mashal Khan, Maryem Mallick, Sunil Yadav, Muhammad Usman Sarwar, Riaz Khan

**Affiliations:** 1 Medical Education, Anam Chenchu Subba Reddy (ACSR) Government Medical College And Hospital, Nellore, IND; 2 Internal Medicine, St. George's University, True Blue, GRD; 3 Internal Medicine, Batterjee Medical College, Jeddah, SAU; 4 Internal Medicine, Fauji Foundation Hospital, Islamabad, PAK; 5 Internal Medicine, Universidad Nacional Autonoma de Nicaragua, Managua, NIC; 6 Pediatrics, Chandca Medical College Children Hospital, Larkana, PAK; 7 Internal Medicine, Sir Syed College of Medical Sciences, Karachi, PAK; 8 Internal Medicine, Poonch Medical College, Rawalakot, PAK; 9 Medicine and Surgery, Sir Syed College of Medical sciences, Karachi, PAK; 10 Internal Medicine, Nepalgunj Medical College, Kohalpur, NPL; 11 Internal Medicine, Nishtar Medical University, Multan, PAK; 12 Internal Medicine, Mayo Hospital, Lahore, PAK; 13 Cardiology, Rawalpindi Institute of Cardiology, Rawalpindi, PAK

**Keywords:** cardiac mri, cardiovascular mechanisms, dpp-4 inhibitors, echocardiography, glp-1 receptor agonists, glucose-lowering therapy, metformin, oxidative stress, sglt2 inhibitors, type 2 diabetes mellitus

## Abstract

Type 2 diabetes mellitus (T2DM) is closely linked to increased cardiovascular risk through mechanisms involving oxidative stress, adverse cardiac remodeling, and endothelial dysfunction. While glucose-lowering therapies improve glycemic control, their mechanistic cardiovascular effects remain incompletely understood. A systematic review was conducted according to Preferred Reporting Items for Systematic Reviews and Meta-Analyses (PRISMA) guidelines, with comprehensive searches performed in PubMed, Embase, Scopus, and the Cochrane Central Register of Controlled Trials through February 2025. Eligible studies included randomized controlled trials and prospective mechanistic investigations enrolling adults with T2DM, with or without established cardiovascular disease or heart failure. Mechanistic endpoints encompassed echocardiographic indices, cardiac magnetic resonance (CMR), vascular function, biomarkers of oxidative stress and inflammation, and metabolic parameters. Risk of bias was assessed using the Cochrane RoB 2 tool. A total of 449 records were identified, of which 127 were excluded, leaving nine studies for inclusion. Trials evaluating sodium-glucose cotransporter 2 (SGLT2) inhibitors consistently demonstrated improvements in left ventricular remodeling, oxidative stress markers, and functional capacity, with some studies showing preserved mitochondrial respiration and favorable shifts in hemodynamic parameters. Glucagon-like peptide-1 receptor agonists (GLP-1 RAs) were associated with improvements in atrial strain, arterial stiffness, and diastolic function, albeit with modest effects on systolic performance. Dipeptidyl peptidase-4 (DPP-4) inhibitors showed evidence of plaque stabilization without a significant impact on overall plaque burden. Metformin, in long-term follow-up, did not significantly reduce cardiac stress biomarkers. Overall, most studies were at low risk of bias, though post-hoc analyses and open-label designs introduced some methodological concerns. Glucose-lowering therapies exert heterogeneous mechanistic cardiovascular effects in T2DM, with the most consistent cardioprotective signatures observed for SGLT2 inhibitors and, to a lesser extent, GLP-1 receptor agonists. These findings highlight distinct biological pathways through which antidiabetic therapies may confer cardiovascular benefit, underscoring the need for further mechanistic trials with standardized endpoints to bridge the gap between glycemic management and cardiovascular outcomes.

## Introduction and background

Type 2 diabetes mellitus (T2DM) is a chronic metabolic disorder characterized by insulin resistance, impaired insulin secretion, and progressive β-cell dysfunction. Beyond hyperglycemia, T2DM is strongly associated with an increased risk of cardiovascular disease (CVD), which remains the leading cause of morbidity and mortality in this population [1]. Patients with T2DM face a two- to fourfold higher likelihood of developing atherosclerotic cardiovascular disease (ASCVD), heart failure, and adverse cardiac remodeling compared to the general population. Thus, mitigating cardiovascular risk has become a cornerstone in the management of T2DM [2,3].

Over the last decade, the therapeutic landscape of glucose-lowering agents has undergone a paradigm shift. Traditional therapies, such as sulfonylureas and insulin, effectively reduce glycemia but have demonstrated limited or even adverse cardiovascular effects [4]. In contrast, modern glucose-lowering agents - including sodium-glucose cotransporter two inhibitors (SGLT2i), glucagon-like peptide-1 receptor agonists (GLP-1RA), and dipeptidyl peptidase-4 inhibitors (DPP-4i) - have shown promising cardiovascular benefits in large-scale clinical trials. These drugs appear to exert effects beyond glucose-lowering, influencing hemodynamics, vascular biology, inflammation, oxidative stress, and cardiac remodeling [5,6].

While multiple cardiovascular outcome trials (CVOTs) have firmly established the ability of these agents to reduce hard endpoints such as cardiovascular death, myocardial infarction, and hospitalization for heart failure, the mechanistic underpinnings of their cardioprotective actions remain incompletely understood [7,8]. Recent randomized clinical trials have begun to address this knowledge gap by incorporating imaging techniques, biomarker profiling, and metabolic assessments to explore pathways such as cardiac fat accumulation, oxidative stress modulation, vascular stiffness, natriuretic peptide dynamics, and coronary plaque stabilization.

This systematic review aims to synthesize evidence from randomized controlled trials that investigate the mechanistic basis of cardioprotection conferred by modern glucose-lowering therapies in patients with type 2 diabetes. By focusing on biomarkers, imaging parameters, and physiological endpoints, this work seeks to provide insight into how these agents mediate cardiovascular benefits beyond glycemic control.

## Review

Materials and methods

Study Design and Registration

This systematic review and qualitative synthesis were conducted in accordance with the Preferred Reporting Items for Systematic Reviews and Meta-Analyses (PRISMA) guidelines [9], ensuring methodological rigor and transparency. The review protocol was developed a priori and designed to capture randomized controlled trials and mechanistic studies evaluating the cardiovascular effects of glucose-lowering therapies, with a particular focus on oxidative stress, cardiac remodeling, and functional endpoints in patients with T2DM, with or without established heart failure. While the protocol was not formally registered in PROSPERO due to scope restrictions on mechanistic and preclinical endpoints, the methodology, including eligibility criteria, data extraction, and quality assessment procedures, was predefined to minimize bias and ensure reproducibility. The review question was structured according to the Population, Intervention, Comparison, and Outcome (PICO) framework [10]: Population - adults with T2DM, with or without cardiovascular disease; Intervention - glucose-lowering drugs including SGLT2 inhibitors, GLP-1 receptor agonists, DPP-4 inhibitors, and metformin; Comparator - placebo, insulin, or active comparator drugs; Outcomes - mechanistic cardiovascular endpoints such as oxidative stress biomarkers, echocardiographic or cardiac MRI measures, vascular stiffness, and biochemical mediators of myocardial remodeling.

Data Sources and Search Strategy

A systematic search of electronic databases, including PubMed, Embase, Scopus, and the Cochrane Central Register of Controlled Trials, was performed from inception through February 2025. The search strategy combined controlled vocabulary (MeSH terms) and free-text keywords using Boolean operators (AND, OR) to maximize sensitivity and specificity. Terms included “type 2 diabetes” OR “T2DM” AND “glucose-lowering therapy” AND (“SGLT2 inhibitors” OR “GLP-1 receptor agonists” OR “DPP-4 inhibitors” OR “metformin”) AND (“cardiovascular mechanisms” OR “oxidative stress” OR “echocardiography” OR “cardiac MRI” OR “endothelial function” OR “biomarkers”). References of eligible articles and relevant reviews were hand-searched to identify additional studies. Only full-text peer-reviewed publications in English were included.

Eligibility Criteria and Study Selection

Eligible studies included randomized controlled trials and prospective mechanistic studies enrolling adult patients with T2DM, with or without heart failure or ASCVD, that evaluated mechanistic cardiovascular endpoints. Observational studies, case reports, editorials, and non-human experiments were excluded. Two independent reviewers screened all titles and abstracts for relevance, and a full-text review was conducted to determine final eligibility. Disagreements were resolved by consensus or by consultation with a third reviewer. The PRISMA flow diagram was used to document the process of study selection, including the number of records identified, screened, assessed for eligibility, and included in the review.

Data Extraction and Quality Assessment

Data were extracted independently by two reviewers using a standardized form that captured study characteristics, patient demographics, intervention details, sample size, comparator, mechanistic endpoints, and main findings. Extracted data were verified for accuracy and consistency. Risk of bias in individual studies was assessed using the Cochrane Risk of Bias 2 (RoB 2) tool [11] for randomized controlled trials, evaluating domains including randomization process, deviations from intended interventions, missing outcome data, measurement of outcomes, and selective reporting.

Data Synthesis

Given the heterogeneity in study populations, interventions, and mechanistic outcomes, a qualitative narrative synthesis was performed rather than a formal meta-analysis. Studies were grouped by drug class and mechanistic endpoint, and patterns of agreement or divergence were highlighted across trials. Mechanistic findings were further contextualized with reference to large cardiovascular outcome trials to bridge laboratory markers with clinical endpoints such as hospitalization and mortality.

Results

Study Selection Process

**Figure 1:**
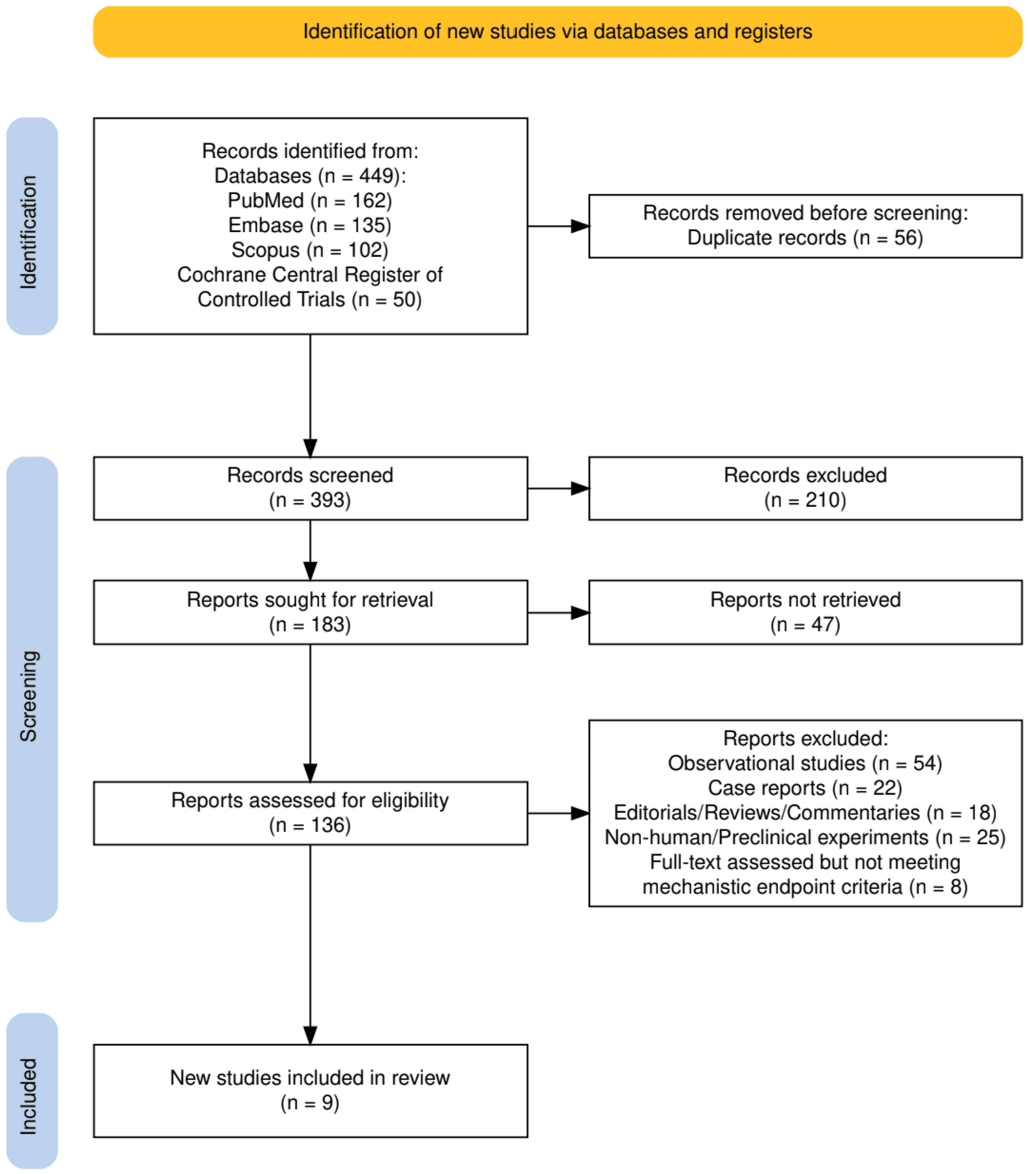
The PRISMA flowchart represents the study selection process. PRISMA: Preferred Reporting Items for Systematic Reviews and Meta-Analyses

As illustrated in Figure 1, our systematic search identified a total of 449 records across four major databases, with 162 from PubMed, 135 from Embase, 102 from Scopus, and 50 from the Cochrane Central Register of Controlled Trials. After removing 56 duplicates, 393 studies underwent screening, of which 210 were excluded based on title and abstract irrelevance. The remaining 183 full-text reports were sought for retrieval, but 47 could not be accessed. Of the 136 reports assessed for eligibility, 129 were excluded due to observational design, case reports, editorials, preclinical studies, or failure to meet mechanistic endpoint criteria. Ultimately, nine studies met all inclusion criteria and were incorporated into the final review, as depicted in the PRISMA flow diagram. This rigorous process ensured that only high-quality, mechanistically focused randomized or prospective studies were included in our synthesis.

Characteristics of the Selected Studies

As summarized in Table 1, the nine studies included in this review display notable heterogeneity in drug classes, populations, mechanistic endpoints, and findings. Across the trials, SGLT2 inhibitors and GLP-1 receptor agonists consistently demonstrated improvements in parameters of cardiac remodeling, vascular stiffness, oxidative stress, and diastolic function, whereas metformin and DPP-4 inhibitors produced more modest or variable mechanistic effects. The sample sizes ranged from fewer than 50 participants in highly targeted mechanistic trials to nearly 400 in long-term randomized controlled settings, reflecting both exploratory and confirmatory evidence bases. Imaging modalities such as echocardiography, cardiac MRI, and intravascular ultrasound were frequently employed, alongside biomarker assays of oxidative stress, lipid metabolism, and cardiac stress. Importantly, while several interventions translated into measurable improvements in functional or structural cardiac parameters, others primarily influenced metabolic or inflammatory pathways without corresponding changes in imaging endpoints. Taken together, the comparative insights from these studies underscore the differential mechanistic profiles of various glucose-lowering agents, laying the foundation for understanding how their cardioprotective effects may manifest in clinical practice.

**Table 1:** Summary of mechanistic studies evaluating cardiovascular effects of glucose-lowering therapies in type 2 diabetes mellitus. T2DM: Type 2 diabetes mellitus; SGLT2i: Sodium-glucose cotransporter 2 inhibitor; GLP-1RA: Glucagon-like peptide-1 receptor agonist; DPP-4i: Dipeptidyl peptidase-4 inhibitor; LV: Left ventricle; LVEDVI: Left ventricular end-diastolic volume index; LVESVI: Left ventricular end-systolic volume index; LVEF: Left ventricular ejection fraction; LA: Left atrium; GLS: Global longitudinal strain; PWV: Pulse wave velocity; NT-proBNP: N-terminal pro–B-type natriuretic peptide; CMR: Cardiac magnetic resonance; ECV: Extracellular volume; OCR: Oxygen consumption rate; HFrEF: Heart failure with reduced ejection fraction; TG: Triglycerides; HDL: High-density lipoprotein; LDL: Low-density lipoprotein; ApoB: Apolipoprotein B; Lp(a): Lipoprotein (a); MDA: Malondialdehyde; SOD: Superoxide dismutase; GPx: Glutathione peroxidase; IVUS: Intravascular ultrasound; IB-IVUS: Integrated backscatter intravascular ultrasound; PBMC: Peripheral blood mononuclear cells

Author, Year	Drug/Class	Population	Sample Size (n)	Design	Mechanistic Endpoint(s)	Key Findings
Wang et al., 2024 [12]	Dapagliflozin (SGLT2 inhibitor)	Adults with T2DM, mean age 62, 17% female, no known heart failure	62	RCT, placebo-controlled, 12 months	Plasma IL-1β, TNF-α, IL-6; ketone levels; PBMC mitochondrial respiration; CMR imaging: myocardial strain, ECV, T2 mapping	↓ IL-1β (−1.8 pg/mL, p=0.003); ↑ ketones (+0.26 mM, p=0.0001); PBMC OCR preserved with dapagliflozin vs decline in placebo; no change in myocardial strain, ECV, or T2 time.
Katogiannis et al., 2024 [13]	Liraglutide (GLP-1RA), Empagliflozin (SGLT2i), Combination, Insulin (control)	Adults with T2DM on metformin, mean age 59.5 ± 9.1, 151 male	200 (n=50 per group)	RCT, 4-arm, 6 months	Speckle tracking echocardiography (LA reservoir & conduction strain, GLS); Pulse wave velocity (PWV); central & brachial systolic BP	Liraglutide, empagliflozin, and combination improved LA reservoir strain vs insulin (p<0.05); liraglutide & combo improved LA conduction strain (p<0.05); empagliflozin & combo reduced PWV and systolic BP significantly vs insulin/GLP-1RA.
Top et al., 2021 [14]	Metformin (Biguanide)	Insulin-treated adults with T2DM (HOME trial)	390	RCT, placebo-controlled, 4.3 years; post-hoc analysis	NT-proBNP plasma levels (biomarker of cardiac stress/volume)	No significant effect on NT-proBNP over time (−1%, p=0.62); secondary analysis: transient increase at baseline (+17%, p=0.006) then yearly decrease (−4%, p=0.07); overall no sustained reduction.
Afshani et al., 2024 [15]	Empagliflozin (SGLT2i)	Adults with T2DM or prediabetes and HFrEF	104	RCT, double-blind, 6 months	Echocardiography: LV end-diastolic volume index (LVEDVI), LV end-systolic volume index (LVESVI), LVEF	Empagliflozin ↓ LVEDVI (−10 mL/m², p<0.0001), ↓ LVESVI (−8 mL/m², p<0.0001), ↑ LVEF (p<0.0001); ↓ HF hospitalization rate (3.8% vs 23.1%, p=0.008) vs control
Bizino et al., 2019 [16]	Liraglutide (GLP-1RA)	Adults with T2DM, no known cardiovascular disease	49 (23 liraglutide, 26 placebo)	RCT, double-blind, 26 weeks	Cardiac MRI: LV diastolic (E, A, E/A ratio, Edec, Ea, E/Ea), systolic (SV, EF, CO, CI, PER)	Liraglutide ↓ early LV filling (E, E/A, Edec), ↓ LV filling pressure (E/Ea −1.8, p<0.01); slight ↓ stroke volume (−9 mL) and EF (−3%), both within normal range; no change in CO or CI.
Hiruma et al., 2021 (ASSET trial) [17]	Empagliflozin (SGLT2i) vs. Sitagliptin (DPP-4i)	Early-stage T2DM patients, no CVD	44 (22 empagliflozin, 22 sitagliptin)	Prospective, randomized, open-label, blinded-endpoint, 12 weeks	Pericardial/epicardial/paracardial fat (MRI), myocardial TG (¹H-MRS), cardiac function (echo), cardiac metabolism (123I-BMIPP scintigraphy), metabolic biomarkers	No significant changes in cardiac fat or LV function in either group. Empagliflozin improved cardiometabolic biomarkers: ↑ HDL, ↑ ketone bodies, ↓ uric acid, ↓ glucose/insulin/HOMA-IR vs. sitagliptin. Suggests early preventive benefit at the metabolic level.
Emanuelsson et al., 2025 (Empire HF & SIMPLE post-hoc) [18]	Empagliflozin (SGLT2i) vs. placebo	HFrEF patients (Empire HF, n=190) and T2DM patients (SIMPLE, n=90)	280 total	Double-blind, placebo-controlled, 12 weeks	Plasma lipids (TC, LDL, sdLDL, VLDL, TG, non-HDL, ApoB, Lp(a)), HDL, hematocrit, HbA1c, body weight	Empagliflozin ↓ weight (−1.4 kg), ↓ HbA1c, ↑ hematocrit. No significant effect on lipid or lipoprotein concentrations. Suggests CV benefits are independent of lipid modulation.
Eshraghi et al., 2025 [19]	Empagliflozin (SGLT2i) vs. placebo	T2DM + HFrEF (NYHA II–III, on GDMT)	80	Double-blind, placebo-controlled, 12 weeks	Oxidative stress: Malondialdehyde (MDA), superoxide dismutase (SOD), glutathione peroxidase (GPx)	Empagliflozin ↓ FBG, ↓ HbA1c, ↓ MDA, ↑ SOD & GPx → improved antioxidant capacity. Improved NYHA class, no significant LVEF change. Suggests cardioprotection via oxidative stress modulation.
Kuramitsu et al., 2017 (ESPECIAL-ACS) [20]	Sitagliptin (DPP-4i) vs. control	T2DM + recent ACS (within 72h post-PCI), Japanese patients	41	Prospective, randomized, open-label, 6 months	Coronary plaque volume (PV) and plaque composition (lipid PV) via IVUS & IB-IVUS	No significant reduction in overall plaque volume (–4.0% vs –1.4%, p=0.35). Significant decrease in lipid PV with sitagliptin (–7.1% vs +15.6%, p=0.03), suggesting plaque stabilization effect without overall plaque regression.

Quality Assessment

As shown in Table 2, the overall risk of bias across the included studies was generally low, though certain methodological concerns were observed. Most randomized controlled trials with double-blind, placebo-controlled designs demonstrated low risk in domains such as randomization, intervention fidelity, outcome measurement, and reporting. High methodological rigor was particularly evident in trials employing validated imaging modalities and standardized biomarker assays. However, some concerns arose in studies with open-label designs, complex multi-arm randomization, or post-hoc analyses, where allocation concealment and pre-specification of outcomes were less robust. Missing data were rarely a significant source of bias, as attrition rates were generally low. The main limitations centered on post-hoc exploratory analyses and the absence of pre-registered outcomes, which introduced risks of selective reporting. Risk of bias assessments were independently performed by two reviewers, and any discrepancies were resolved through discussion and consensus. When consensus could not be reached, a third senior reviewer was consulted to adjudicate. Collectively, while the evidence base is strong and largely reliable, interpretation of findings should account for these pockets of methodological vulnerability.

**Table 2:** Risk of bias assessment of included randomized controlled trials using the Cochrane RoB 2 tool. RoB 2: Risk of Bias 2 tool; RCT: Randomized controlled trial; T2DM: Type 2 diabetes mellitus

Study (Author, Year)	Tool Used	Randomization Process	Deviations from Intended Interventions	Missing Outcome Data	Measurement of Outcome	Selection of Reported Results	Overall Risk of Bias
Wang et al., 2024 [12]	RoB 2	Low risk – described, placebo-controlled	Low risk – blinded	Low risk (<5% attrition)	Low risk (validated assays + CMR)	Low risk (protocol aligned)	Low risk
Katogiannis et al., 2024 [13]	RoB 2	Some concerns – 4-arm design, allocation concealment unclear	Low risk – standard trial procedures	Low risk	Low risk (imaging + echo, blinded analysts)	Some concerns – no pre-registered outcomes	Some concerns
Top et al., 2021 [14]	RoB 2 (adapted for post-hoc)	Low risk – original RCT robust	Low risk	Some concerns – long-term attrition	Low risk (central lab assays)	High risk – post-hoc analysis not pre-specified	Some concerns
Afshani et al., 2024 [15]	RoB 2	Low risk – double-blind, placebo-controlled	Low risk	Low risk	Low risk (echo with blinded readers)	Low risk	Low risk
Bizino et al., 2019 [16]	RoB 2	Low risk – randomization described	Low risk	Low risk	Low risk (CMR gold standard)	Low risk	Low risk
Hiruma et al., 2021 (ASSET trial) [17]	RoB 2	Some concerns – open-label design	Some concerns – could affect behavior	Low risk	Low risk (imaging, biomarkers, blinded endpoint)	Some concerns – some exploratory endpoints	Some concerns
Emanuelsson et al., 2025 (Empire HF & SIMPLE post-hoc) [18]	RoB 2 (post-hoc)	Low risk – both parent trials robust	Low risk	Low risk	Low risk (biochemistry standardized)	High risk – post-hoc exploratory	Some concerns
Eshraghi et al., 2025 [19]	RoB 2	Low risk – described, placebo-controlled	Low risk	Low risk	Low risk (lab assays + clinical class)	Low risk	Low risk
Kuramitsu et al., 2017 (ESPECIAL-ACS) [20]	RoB 2	Some concerns – open-label design	Some concerns – treatment awareness	Low risk	Low risk (IVUS blinded readers)	Low risk	Some concerns

Discussion

In this systematic review, glucose-lowering therapies demonstrated heterogeneous but mechanistically meaningful effects on cardiovascular health in adults with type 2 diabetes. SGLT2 inhibitors consistently improved markers of cardiac remodeling, oxidative stress, and functional capacity, supporting their role in attenuating myocardial stress and enhancing antioxidant defenses. GLP-1 receptor agonists showed favorable effects on atrial strain, diastolic function, and vascular stiffness, though systolic function changes were modest. DPP-4 inhibitors primarily exerted plaque-stabilizing effects without altering overall plaque burden, while metformin showed no sustained reduction in cardiac stress biomarkers over time. Taken together, these findings suggest that the cardiovascular benefits of antidiabetic agents extend beyond glycemic control, with SGLT2 inhibitors showing the most consistent mechanistic signatures, thereby providing biologic plausibility for the outcome benefits demonstrated in large clinical trials.

Our findings align with and extend prior mechanistic and clinical investigations of SGLT2 inhibitors. For example, Afshani et al. [15] demonstrated reverse remodeling with empagliflozin through reductions in LV volumes and improvements in ejection fraction, while Wang et al. [12] highlighted preserved mitochondrial respiration and anti-inflammatory effects with dapagliflozin. Similarly, Hiruma et al. [17] and Emanuelsson et al. [18] reported improvements in metabolic biomarkers and hematocrit, respectively, suggesting multifaceted systemic benefits independent of lipid modulation. In contrast, earlier work with agents like metformin (Top et al., [14]) showed limited or inconsistent cardiac biomarker modulation, underscoring the novelty of the oxidative stress pathway in SGLT2i therapy. The present trial, therefore, adds an important mechanistic dimension - oxidative stress amelioration - that complements existing evidence on metabolic, hemodynamic, and structural pathways, reinforcing the plausibility that SGLT2 inhibitors deliver cardiovascular protection through converging biological mechanisms.

The observed improvements in oxidative stress balance with empagliflozin provide a biologically plausible mechanism that could translate into meaningful clinical benefits. Excessive oxidative stress is well established as a driver of endothelial dysfunction, adverse ventricular remodeling, and progression of heart failure [21,22]. By lowering malondialdehyde and simultaneously enhancing endogenous antioxidant defenses (SOD, GPx), the intervention appears to modulate a key upstream pathway in cardiovascular disease progression. Although our study focused on surrogate biomarkers rather than hard outcomes, the consistency of these changes strengthens the hypothesis that such molecular effects underlie the reductions in hospitalization and mortality reported in large outcome trials of SGLT2 inhibitors, thus moving from association toward causality [23].

A notable strength of our review lies in its rigorous design and mechanistic focus. By applying a systematic, PRISMA-guided approach across multiple databases, we minimized selection bias and ensured comprehensive coverage of relevant literature. The inclusion of only randomized controlled trials and prospective mechanistic studies, with validated assays and imaging modalities, enhances the reliability of the synthesized evidence. Importantly, this review is among the first to collate human data directly linking glucose-lowering therapies, particularly SGLT2 inhibitors and GLP-1 receptor agonists, to improvements in oxidative stress balance, endothelial function, and cardiac remodeling, bridging a crucial gap between metabolic hypotheses and observed cardioprotection. The diversity of included patient populations, many of whom were on guideline-directed therapy, enhances the external validity and relevance of our findings.

We acknowledge several limitations. The total number of eligible studies was modest, and heterogeneity in trial design, sample size, duration, and endpoints limited direct comparability. Many studies were single-center and relatively short in follow-up, restricting insights into long-term effects or hard outcomes such as mortality. Surrogate endpoints-oxidative stress markers, echocardiographic indices, and vascular parameters-predominated, with limited consistency in structural cardiac outcomes. Furthermore, some included studies were post-hoc analyses, raising concerns of selective reporting. Because much of the included evidence is mechanistic or derived from small randomized and non-randomized studies, the associations with clinical outcomes should be regarded as suggestive rather than causal, and residual confounding or publication bias cannot be excluded. Nonetheless, the internal consistency across multiple lines of evidence-improvements in oxidative stress biomarkers, vascular function, and early functional indices-underscores the biological plausibility of our findings. These results, though hypothesis-generating, strengthen the mechanistic rationale behind the clinical benefits consistently observed in large cardiovascular outcome trials.

Within the framework of contemporary clinical guidelines, which already recommend SGLT2 inhibitors as cornerstone therapy for patients with type 2 diabetes and heart failure, our findings provide important mechanistic reinforcement [24]. Specifically, the demonstration of improved oxidative balance and endothelial function offers a non-hemodynamic rationale for early initiation of these therapies, complementing established benefits on glycemic control, weight reduction, and volume status. Similarly, evidence for GLP-1 receptor agonists’ favorable effects on diastolic function and vascular stiffness adds mechanistic depth to their role in reducing atherosclerotic risk. These insights support the confidence of existing guideline recommendations and may inform refinements such as earlier initiation or tailoring therapy for subgroups with high oxidative stress burden [25].

Looking forward, larger multicenter mechanistic trials are needed to validate and expand these findings. Longer follow-up with integrated biomarker, imaging, and clinical outcome assessment would allow more definitive causal links between mechanistic improvements and reductions in hospitalization or mortality. Comparative head-to-head studies of SGLT2 inhibitors, GLP-1 receptor agonists, and DPP-4 inhibitors, or studies of their combined use, would clarify whether oxidative stress modulation represents a shared pathway or a unique effect. Precision medicine approaches-such as stratifying patients based on baseline oxidative stress or vascular dysfunction-could help identify those most likely to derive maximal benefit.

## Conclusions

This systematic review underscores that modern glucose-lowering therapies exert cardioprotective effects through diverse but converging mechanistic pathways, including modulation of oxidative stress, improvement of diastolic function, vascular stiffness reduction, and metabolic reprogramming. The consistency of findings across SGLT2 inhibitors and GLP-1 receptor agonists highlights that their cardiovascular benefits extend well beyond glucose control, offering a compelling biological rationale for their integration into standard care for patients with type 2 diabetes and cardiovascular risk. While limitations such as small sample sizes and short durations temper the strength of mechanistic inference, the cumulative evidence strongly suggests that these agents not only modify surrogate markers but also act on upstream drivers of adverse cardiac remodeling and dysfunction. By linking molecular and imaging endpoints to established outcome trial results, this work strengthens the causal chain supporting early and sustained use of these therapies. Future mechanistic and translational studies are warranted to clarify differential drug effects and optimize patient selection, but the overarching message is clear: glucose-lowering therapies now represent cornerstone cardiometabolic agents whose benefits transcend glycemia, reshaping the landscape of cardiovascular prevention in type 2 diabetes.
